# MEASUREMENT CHALLENGES AND SOLUTIONS WHEN EVALUATING PHYSICAL ACTIVITY AMONG HOSPITALIZED PATIENTS WITH DEMENTIA

**DOI:** 10.1093/geroni/igac059.1535

**Published:** 2022-12-20

**Authors:** Brittany Drazich

**Affiliations:** University of Maryland, Baltimore, Maryland, United States

## Abstract

Obtaining subjective reports of physical activity from individuals is known to be biased. With increased creation, dissemination, and use of technology, approaches for objective measurement have improved. Although actigraphy is increasingly used, challenges include a lack of clearly established cut off levels for vigorous, moderate, and low level activity in older individuals, decreased arm movement with walking, and the impact of comorbidities (e.g., Parkinson’s Disease) on actigraphy readings. The advantages of the MotionWatch 8 over other devices will be discussed and our protocol for use shared. The findings from the first 200 participants will be described and challenges to interpretation addressed. The majority of the study participants were willing to wear the MotionWatch 8 during hospitalization. Using innovative approaches to avoid direct patient contact due to concerns related to COVID-19, follow up activity was obtained at 1 month post discharge. Lastly, recommendations for interpretation of findings will be provided.

